# Efforts to prevent surgical sponge retention: check X-ray on the big screen

**DOI:** 10.1186/s40981-025-00780-1

**Published:** 2025-03-14

**Authors:** Kengo Ito, Shotaro Ishimoto, Chiaki Nemoto, Keisuke Yoshida, Satoki Inoue

**Affiliations:** 1 The Junior Resident Center, Ohara General Hospital, 6-1 Ohomachi, Fukushima, 960-8611 Japan; 2 Department of Anesthesiology, Ohara General Hospital, 6-1 Ohomachi, Fukushima, 960-8611 Japan; 3 Department of Anesthesiology, Fukushima Medical University, 1 Hikarigaoka, Fukushima, 960-1295 Japan

To the Editor,

Retained surgical items (RSIs), most often surgical sponges, may occur as a result of human error. If RSIs go undetected, patients may develop gossypibomas several years or even decades later [1]. Although postoperative radiographs are now widely obtained in the operating room to detect RSIs, these items may remain undetected. Factors contributing to RSIs vary depending on the type of surgery, surgical site, a patient’s physical characteristics, and whether the surgery is an emergency or scheduled [2, 3]. It is difficult to completely prevent RSIs by relying solely on human effort [4]. However, clinically, the standard method still relies on human verification through radiographs to confirm RSIs, in addition to counting these items.

Recently, we encountered a case in which the sponge count did not match during a previous cesarean section, and a gossypiboma was diagnosed during a second cesarean Sect. 2 years later, after reviewing radiographic images obtained at the first surgery (Fig. 1).

**Fig. 1:**
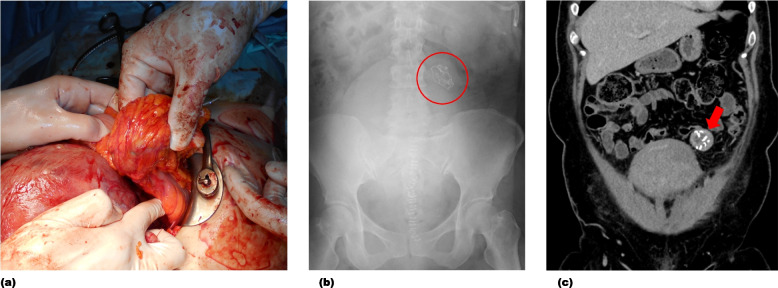
Findings in a patient with a retained surgical item. **a** A mass, which appeared to be of mesenteric origin and measured approximately 4 cm, was observed above the uterus during a cesarean section performed 2 years after a previous cesarean section. **b** Postoperative radiographs showing a suspected retained surgical sponge during the current cesarean section. However, because the sponge counts were correct, the current surgery was completed. **c** Computed tomography performed 2 days after the current cesarean section revealed a retained surgical sponge from the first cesarean section, which required surgical removal

Although we cannot depend completely on artificial intelligence to prevent RSIs, these tools may be appropriate in RSI diagnostic imaging to prevent human error [5]. However, this approach will involve time and expense to implement. Other methods are needed in the meantime. It is practically impossible for a radiologist to devote time solely to confirm RSIs. Since this incident, we have made it a practice to have the radiographs regularly reviewed by the surgeons in charge as well as all staff involved in the surgery: surgeons, anesthesiologists, nurses, and radiographers. Additionally, instead of reviewing the radiographs on a small portable screen, we now ensure they are reviewed by all staff on a large screen in the operating theater (Fig. 2). Although the surgeon is ultimately responsible for RSIs, we believe it is meaningful to check for RSIs and share the images with all staff members to help eliminate RSIs. Importantly, because image resolution on both screens is the same, an abnormality that is invisible on the small screen may also be invisible on the big screen. However, when in doubt, the images can be rechecked by adjusting the brightness and contrast. While it is ideal for all team members to check for RSIs after surgery, in situations such as late-night emergency surgeries with limited staff, the anesthesiologist may also play a crucial role in ensuring this process is completed reliably, with the surgeons. However, even if these checks are performed properly when multiple staff members check radiographs, there may be a risk of the responsibility shifting between members, which could inadvertently lower the quality of the analysis [6]. It is also important to create a psychologically safe workplace where all staff can freely share opinions [7].

**Fig. 2:**
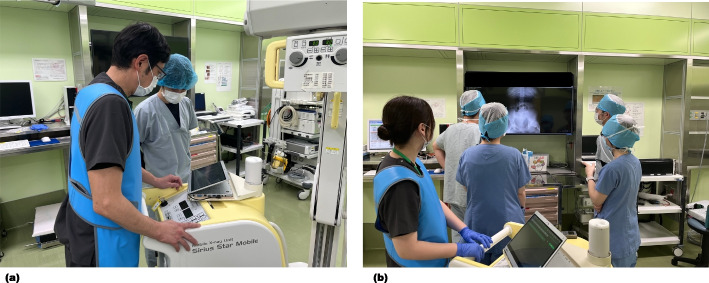
Images of the surgical team checking the radiographs for retained surgical items. **a** A single surgeon checks the image on a small portable screen to ensure there are no retained surgical items. The radiographer is concerned about whether the imaging area is adequate. **b** The radiographs are immediately transferred to the electronic medical record and shared on the big screen so that all staff members can view the images together. All members involved in the surgery check for any abnormalities in the images on the big screen. Because the resolution of the images on both screens is the same, an abnormality that is invisible on the small screen may also be invisible when viewing the image on the big screen. However, when in doubt, the images can be rechecked by adjusting the brightness and contrast

## Limitation

Because the frequency of RSIs is low, we cannot determine whether our current methods are effective.

## Conclusion

RSIs are rare but still occur due to human error, and it may be impossible to eliminate them completely. The future use of artificial intelligence tools may be promising to eliminate RSIs; however, implementation remains difficult. Therefore, involving as many people as possible with the ability to interpret radiographs might help reduce the chance of RSIs. We must be relentless in our efforts to prevent RSIs.

## Data Availability

Not applicable.

## Abbreviation

RSIs: Retained surgical items
